# Supplementary material for: Short- and Long-Term Outcome of Laparoscopic- versus Robotic-Assisted Right Colectomy: A Systematic Review and Meta-Analysis
Source: J Clin Med. 2022 Apr 24;11(9):2387. doi: 10.3390/jcm11092387 (PMC9103048; doi:10.3390/jcm11092387)
Supplement: Supplementary file 1 [file jcm-11-02387-s001.zip › jcm-1676389-supplementary.pdf]

Supplement Table S1: Pathological Staging and Information about adjuvant treatment of studies with a long-term follow up:

**Author**			**TNM: *n* (%)**														**UICC: *n* (%)**										**Adjuvant Treatment**	
	**LRC (n)**	**RRC (n)**	Tis (L)	Tis (R)	T1 (L)	T1 (R)	T2 (L)	T2 (R)	T3 (L)	T3 (R)	T4 (L)	T4 (R)	N0 (L)	N0 (R)	N+ (L)	N+ (R)	0 (L)	0 (R)	I (L)	I (R)	II (L)	II (R)	III (L)	III (R)	IV (L)	IV (R)	(L)	(R)
Ferri [13]	35	35	-	-	7(20)	9(26)	18(51)	17(49)	7(20)	6(17)	-	-	-	-	-	-	-	-	-	-	-	-	-	-	-		n/a	n/a
Park [7, 11]	35	35	-	-	10(29)	9(26)	16(17)	16(46)	9(26)	10(29)	*	*	-	-	-	-	-	-	-	-	-	-	-	-	-		23(66)	21(60)
Spinoglio [8]	100	100	-	-	-	-	-	-	-	-	-	-	-	-	-	-	-	-	26(26)	21(21)	28(28)	38(38)	33(33)	37(37)	13(13)	4(4)	n/a	n/a
Kang [33]	43	20	-	-	7(16)	5(25)	16(37)	7(35)	20(47)	8(40)	-	-	-	-	-	-	-	-	-	-	-	-	-	-	-		n/a	n/a

Values are given in absolute numbers or percentages. Abbreviations: n/a = not available, SD = Standard deviation, f = female, m = male.

Gray: Laparoscopic resections. White: Robotic resections.

*T4 tumors were not included in this study [7,11].

Supplement Table S2A: Pathological TNM Staging if available:

Author		TNM: *n* (%)														
	**LRC (n)**	**RRC (n)**	Tis		T1		T2		T3		T4		N0		N+	
Yozgatli [15]	61	35	3(5)	2(6)	7(11)	5(14)	20(33)	13(20)	29(45)	12(34)	3(5)	2(6)	-	-	-	-
Ferri [13]	35	35	-	-	7(20)	9(26)	18(51)	17(49)	7(20)	6(17)	-	-	-	-	-	-
Park [7, 11]	35	35	-	-	10(29)	9(26)	16(46)	16(46)	9(26)	10(26)	*	*	-	-	-	-
Spinoglio [8]	100	100	-	-	-	-	-	-	-	-	-	-	-	-	-	-
Migliore [14]	170	46	2(1)	1(2)	17(10)	6(13)	31(18)	5(11)	87(51)	26(57)	26(15)	5(11)	111(65)	24(52)	52(31)	19(41)
Hannan [17]	35	35	-	-	5(14)	5(14)	8(23)	1(3)	11(31)	11(31)	4(11)	3(9)	-	-	-	-
Tagliabue [18]	68	55	13(19)	14(25)	11(16)	16(29)	21(31)	14(25)	22(32)	10(18)	1(1)	1(2)	-	-	-	-
Dohrn [10]	3621	381	6(0.1)	1(0.2)	304(8)	38(10)	574(16)	78(20)	2077(57)	194(51)	598(17)	64(17)	2281(63)	251(66)	1281(35)	125(33)
Merola [16]	94	94	-	-	13(14)	10(11)	56(60)	52(55)	23(24)	31(33)	3(3)	2(2)	-	-	-	-
Ahmadi [36]	42	59	7(17)	16(27)	4(10)	8(14)	3(7)	5(8)	19(45)	20(34)	5(12)	8(14)	23(55)	43(73)	14(33)	16(27)
Ngu [20]	16	16	0	1(6)	1(6)	0(0)	2(13)	1(6)	9(56)	10(62)	3(19)	2(13)	9(56)	7(44)	6(38)	7(44)
Sorgato [21]	40	48	2(5)	7(15)	9(23)	18(38)	14(35)	11(23)	9(23)	10(21)	6(15)	2(4)	-	-	-	-
Widmar [19]	207	69	-	-	-	-	-	-	-	-	-	-	-	-	-	-
Gerbaud [22]	59	42	-	-	-	-	-	-	-	-	-	-	-	-	-	-
Mégevand [23]	50	50	-	-	-	-	-	-	-	-	-	-	-	-	-	-
Trastulli [24]	134	102	7(5)	7(7)	33(25)	23(23)	36(27)	26(25)	36(27)	30(30)	8(6)	2(2)	-	-	-	-
Ceccarelli [25]	29	26	5(17)	2(8)	4(14)	1(4)	2(7)	6(23)	17(59)	11(42)	1(3)	6(23)	23(7)	17(65)	6(21)	9(35)
De Angelis [26]	50	30	-	-	18(36)	8(27)	21(42)	13(43)	11(22)	9(30)	-	-	-	-	-	-
Deutsch [27]	47	18	1(2)	0(0)	13(28)	3(17)	6(13)	1(6)	4(9)	1(6)	-	-	-	-	-	-
Haskins [28]	2405	89	-	-	-	-	-	-	-	-	-	-	-	-	-	-
Rawlings [30]	15	17	-	-	-	-	-	-	-	-	-	-	-	-	-	-
deSouza [31]	135	40	6(4)	1(3)	16(12)	3(8)	14(10)	7(18)	19(14)	4(10)	11(8)	3(8)	49(36)	14(35)	17(13)	4(10)
Casillas [32]	110	52	-	-	-	-	-	-	-	-	-	-	-	-	-	-
Kang [33]	43	20	-	-	7(16)	5(25)	16(37)	7(35)	20(47)	8(40)	-	-	-	-	-	-
Dolejs [34]	6521	259	-	-	-	-	-	-	-	-	-	-	-	-	-	-
Lujan [35]	135	89	6(4)	6(7)	28(21)	14(16)	22(16)	10(11)	19(14)	13(14)	6(4)	3(3)	-	-	-	-

Values are given in absolute numbers and percentage.

Gray: Laparoscopic resections. White: Robotic resections.

*T4 was an exclusion criterion in this study.

Supplement Table S2B: Pathological UICC Staging if available:

**Author**			**UICC: *n* (%)**									
	**LRC (n)**	**RRC (n)**	0		I		II		III		IV	
Yozgatli [15]	61	35	-	-	-	-	-	-	-	-	-	-
Ferri [13]	35	35	-	-	-	-	-	-	-	-	-	-
Park [7, 11]	35	35	-	-	-	-	-	-	-	-	-	-
Spinoglio [8]	100	100	-	-	26(26)	21(21)	28(28)	38(38)	33(33)	37(37)	13(13)	4(4)
Migliore [14]	170	46	-	-	-	-	-	-	-	-	-	-
Hannan [17]	35	35	-	-	-	-	-	-	-	-	-	-
Tagliabue [18]	68	55	-	-	-	-	-	-	-	-	-	-
Dohrn [10]	3621	381	5(0.1)	1(0.2)	745(21)	89(23)	1435(40)	143(38)	1114(31)	109(29)	228(6)	33(9)
Merola [16]	94	94	-	-	-	-	-	-	-	-	-	-
Ahmadi [36]	42	59	-	-	-	-	-	-	-	-	-	-
Ngu [20]	16	16	-	-	-	-	-	-	-	-	-	-
Sorgato [21]	40	48	-	-	-	-	-	-	-	-	-	-
Widmar [19]	207	69	-	-	-	-	-	-	-	-	-	-
Gerbaud [22]	59	42	-	-	-	-	-	-	-	-	-	-
Mégevand [23]	50	50	15(30)	9(18)	7(14)	10(20)	9(18)	16(32)	16(32)	12(24)	3(6)	3(6)
Trastulli [24]	134	102	-	-	-	-	-	-	-	-	-	-
Ceccarelli [25]	29	26	-	-	-	-	-	-	-	-	-	-
De Angelis [26]	50	30	-	-	-	-	-	-	-	-	-	-
Deutsch [27]	47	18	-	-	-	-	-	-	-	-	-	-
Haskins [28]	2405	89	-	-	-	-	-	-	-	-	-	-
Rawlings [30]	15	17	-	-	-	-	-	-	-	-	-	-
deSouza [31]	135	40	-	-	-	-	-	-	-	-	-	-
Casillas [32]	110	52	-	-	-	-	-	-	-	-	-	-
Kang [33]	43	20	-	-	-	-	-	-	-	-	-	
Dolejs [34]	6521	259	-	-	-	-	-	-	-	-	-	-
Lujan [35]	135	89	-	-	-	-	-	-	-	-	-	-

Values are given in absolute numbers and percentage.

Gray: Laparoscopic resections. White: Robotic resections.

Supplement Table S3: Meta-analysis of perioperative Outcome of included studies:

**Variable**	**LRC**	**RRC**	**OR/MD**	***p*-Value**	**I²**	**References**
Surgery costs (€)	3.900 ± 1.677	8.156 ± 0.458	-4.16 [-7.12 - -1.21]*	**0.006**	93%	[16, 33]
Total costs (€)	7.647 ± 1.307	10.306 ± 1.507	-2.66 [-5.17 - -0.15]*	**0.04**	96%	[7, 11, 16, 30, 33]

Values are given in mean ±SD or in absolute numbers. Abbreviations: OR: Odds ratio, MD: Mean difference, d: days, ml: milliliter, min: minutes.

*x1000€.
